# Antineutrophil cytoplasmic antibodies and their association with clinical outcomes in hospitalized COVID-19 patients

**DOI:** 10.1038/s41420-021-00671-1

**Published:** 2021-10-05

**Authors:** Kamran Kadkhoda, Kristen Laurita

**Affiliations:** 1 Immunopathology Laboratory, Robert J. Tomsich Pathology & Laboratory Medicine Institute, Cleveland Clinic, Cleveland, OH USA; 2 Cleveland Clinic Lerner College of Medicine, Case Western Reserve University, Cleveland, OH USA

**Keywords:** Antibodies, Viral infection

As indubitably the most catastrophic pandemic of the 21^st^ century so far, coronavirus disease 2019 (COVID-19) has shown many faces. This ranges from direct viral cytopathic effect to triggering immunopathological mechanisms through which COVID-19 can further exert tissue damage. One of the important features is the elevated neutrophil-to-lymphocyte-count ratio, especially during moderate and severe COVID-19 and the elevated circulating neutrophil extracellular traps (NETs) [1–5]. NETs are typically studded with several proteins, including a variety of neutrophils’ enzymes. NETs, in turn, can activate complement pathways further causing tissue destruction and vasculitis. Antineutrophil cytoplasmic antibodies (ANCAs) are a type of autoantibodies that are typically directed against neutrophils’ cationic enzymes such as proteinase 3 (PR3), myeloperoxidase (MPO), elastase, lactoferrin, cathepsin G, bacterial permeability-increasing peptide, and calprotectin. They are involved in the pathomechanism of small-to-medium-sized vessel vasculitides. ANCAs can be detected by indirect immunofluorescence assay (IFA) and are named based on the staining pattern. If the neutrophil cytoplasm is almost evenly stained but the nucleus is not, it is called cytoplasmic ANCA (C-ANCA), whereas when only the nucleus is stained but the cytoplasm is not, it is called perinuclear ANCA (P-ANCA). Although not universal, PR3 is associated with the former and MPO with the latter. C- or P-ANCAs can be by-products of the aforementioned NET-formation mechanism [1]. Although ANCAs are known to play a role in vasculitides generally in the context of autoimmune diseases, their generation can also be elicited by certain infections such as *Staphylococcus aureus* [4].

Given the known pathophysiology of COVID-19 so far, we set out to investigate the prevalence of ANCAs among 100 randomly selected hospitalized patients with confirmed COVID-19 diagnoses in 2020. Blood specimens were drawn from these patients at the Cleveland Clinic hospitals and stored at the biorepository for future studies. Our study was approved by the institutional review board. There were two groups of 50 patients: moderate and severe, the latter was defined as patients with any of intensive care unit (ICU) admission, mechanical ventilation, extracorporeal membrane oxygenation (ECMO), or death, whereas the moderate cases did not meet any of the above-mentioned criteria. Samples were chosen randomly by the institutional biorepository staff based on the requested criteria and then were blinded to the investigators. Samples were collected from 9 to 28 days (median 19.5) post onset of signs and symptoms from patients with a median age of 59 years (range: 21–91). In total, 51 patients were male and the rest were female. Overall, 48% had ICU admission, 31% had mechanical ventilation, none received ECMO, and 15% died. All samples were screened for ANCAs by IFA using ethanol-fixed slides. Given the almost pervasive fluorescence activity among the screen slides, the samples were all also examined for ANCAs using formalin-fixed slides as the confirmatory test. All samples were also tested for anti-PR3 and anti-MPO antibodies using a random-access platform based on multiplex-fluorescence immunoassay (MFIA) principle. The three tests mentioned above are routinely performed in our laboratory.

Overall, 57% of sera tested confirmed positive for ANCAs, of which 72% and 28% were confirmed C-ANCA and P-ANCA, respectively. Only one sample tested positive for anti-MPO antibody; three other samples with matching ANCA results had antibody-index values of 0.4–0.6 range (cutoff: 1.0), suggesting some had weak reactivity below the cutoff. Of 15 patients who died, 17.5% had confirmed ANCA results compared with 11.6% of survivors with no confirmed ANCA result, though this did not reach statistical significance. Furthermore, 63.4% of severe cases had confirmed C-ANCA results compared with 34.9% of patients with no confirmed ANCAs (OR: 1.81, 95% CI: 0.86–3.75; *P* = 0.13, Fisher exact). There was no association between age and sex with ANCA positivity. A subtle, but nonsignificant trend, was noticed with P-ANCA positivity with increasing age. Most interestingly, there was a strong association between confirmed C-ANCA status with ICU admission (OR: 3.250, 95% CI: 1.31–7.60, *P* = 0.0087) in contrast to the confirmed P-ANCA status, whereas this association was marginal for mechanical ventilation (OR: 3; 95% CI: 1.08–8.67, *P* = 0.05) (Table 1).

**Table 1:** Antineutrophil cytoplasmic antibodies and their association with clinical outcomes in hospitalized COVID-19 patients.

Age at specimen collection (years)	21–91 (median: 59)		
Female sex	49/100		
Specimen collection relative to onset (days)	9–28 (median 19.5)		
Severe COVID-19	50/100		
ICU admission	48/100		
Mechanical ventilation	31/100		
ECMO	0/100		
Death	15/100		
Total confirmed ANCA	57/100		
Confirmed C-ANCA	41/100		
Confirmed P-ANCA	16/100		
Confirmed ANCA in			
Death	10/15 47/85	OR: 1.20; 95%CI: 0.49–2.89	*P* value = 0.65 (Fisher exact, GraphPad Prims 9)
Survival			
Severity in relation to C-ANCA status			
Confirmed	26/41 (63.4%) 15/43 (36.6%)	OR: 1.81; 95%CI: 0.86–3.75	*P* value = 0.13 (Fisher exact, GraphPad Prims 9)
Not confirmed			
Sex in relation to confirmed ANCA status			
Male	29/51 (57%) 28/49 (57%)	OR: 1.00; 95%CI: 0.51–1.95	*P* value > 0.99 (Fisher exact, GraphPad Prims 9)
Female			
Age at specimen collection in relation to ANCA status			*P* value = 0.50 (Mann–Whitney U test, GraphPad Prims 9)
Confirmed (median) Not Confirmed (median)	61 years 58 years		
ICU admission in relation to conformed C-ANCA status		OR: 3.250; 95%CI: 1.31–7.60	*P* value = 0.0087 (Fisher exact, GraphPad Prims 9)
Confirmed Not conformed	26/48 (54.1%) 8/48 (16.6%)		
Mechanical ventilation in relation to conformed C-ANCA status			
Confirmed	18/31 (58%) 6/31 (19.3%)	OR: 3.0; 95%CI: 1.08–8.67	*P* value = 0.05 (Fisher exact, GraphPad Prims 9)
Not conformed			

Our finding of such high prevalence of ANCAs among hospitalized patients was unexpected and very exciting. To the best of our knowledge, this is the first time that such study is done in these patients, especially using IFA for ANCAs. As mentioned earlier, there are a number of neutrophil enzymes that can trigger ANCAs, but with the exception of PR3 and MPO, they are not tested for using current immunoassays such as MFIA that was used here. In fact, when NETs are produced, they are decorated with these enzymes, so under natural circumstances they can destroy microorganisms such as bacteria that are stuck in these NETs. Since this is a local reaction, the nearby endothelial cells are damaged not only directly by these detrimental enzymes but also through triggering immunothrombosis by these enzymes. The latter can be particularly problematic in tissues with large vasculature such as lungs, leading to pulmonary embolism. Through production of C5a, complement activation due to the presence of NETs attracts more neutrophils to the site, further exacerbating the situation.

Since we did not have samples available before the onset of COVID-19 or after hospital discharge, we could not follow up on the ANCA status over time. It is important to highlight that both ANCA and MPO/PR3 tests detected IgG antibodies, thus, we could not rule out the presence of IgM antibodies, given the short period after COVID-19 onset, hence little time for a class-switched autoimmune response to fully develop. This means the several screen-positive-only sera would have possibly been confirmed had the study included specimens drawn several weeks or months after onset. Of note, that corticosteroids are used for managing COVID-19 patients on mechanical ventilation or in the ICU, and are also used routinely for managing patients with ANCA-associated vasculitides, points to a possible shared pathomechanism. This study calls for longitudinal follow-ups in patients with a history of confirmed COVID-19 diagnosis who have been experiencing autoimmune phenomena suggestive of vasculitis. Routinely investigating ANCA among hospitalized COVID-19 patients, especially shortly after admission, may serve as a biomarker to predict worse outcomes. This notion needs substantiating through longitudinal studies.

## Supplementary information

APC Waiver

## Data Availability

Data are available to the journal and the publisher upon request.
