# Supplementary material for: Antineutrophil cytoplasmic antibodies and their association with clinical outcomes in hospitalized COVID-19 patients
Source: Cell Death Discov. 2021 Oct 5;7:277. doi: 10.1038/s41420-021-00671-1 (PMC8491172; doi:10.1038/s41420-021-00671-1)
Supplement: Supplementary file 1 — APC Waiver [file 41420_2021_671_MOESM1_ESM.pdf]

Cleveland Clinic
Robert J. Tomsich Pathology & Laboratory Medicine Institute

David Bosler, MD
Chair
Department of Laboratory Medicine

September 1, 2021

To Whom It May Concern:

This letter confirms that Kamran Kadkhoda, PhD does not have any research grants that cover article processing fees or publication costs.

Sincerely,

David Bosler, MD

/jc

Cleveland Clinic
10300 Carnegie Ave., LL2-137
Cleveland, Ohio 44106

Tel 216 636 9615
boslerd@ccf.org
